# The DEVD motif of Crimean-Congo hemorrhagic fever virus nucleoprotein is essential for viral replication in tick cells

**DOI:** 10.1038/s41426-018-0192-0

**Published:** 2018-11-28

**Authors:** Cristiano Salata, Vanessa Monteil, Helen Karlberg, Michele Celestino, Stephanie Devignot, Mikael Leijon, Lesley Bell-Sakyi, Éric Bergeron, Friedemann Weber, Ali Mirazimi

**Affiliations:** 1 Department of Molecular Medicine, University of Padova, IT-35121 Padova, Italy; 2 Department of Microbiology, Public Health Agency of Sweden, SE-171 82 Solna, Sweden; 3 Department of Laboratory Medicine, Karolinska University Hospital and KI, SE-14186 Huddinge, Stockholm Sweden; 4 Institute for Virology, FB10-Veterinary Medicine, Justus Liebig University Giessen, DE-35392 Giessen, Germany; 5 National Veterinary Institute, SE-756 51 Uppsala, Sweden; 6 Department of Infection Biology, Institute of Infection and Global Health, University of Liverpool, Liverpool, L3 5RF UK; 7 Viral Special Pathogens Branch, Division of High-Consequence Pathogens and Pathology, National Center for Emerging and Zoonotic Infectious Diseases, Centers for Disease Control and Prevention, Atlanta, GA 30333 USA

Dear Editor,

Crimean-Congo hemorrhagic fever (CCHF) is an emerging tick-borne viral disease widely distributed across countries of Africa, Southern Europe, the Middle East, and Asia^1^. CCHF is caused by Crimean-Congo hemorrhagic fever virus (CCHFV; genus *Orthonairovirus*, family *Nairoviridae)*, which usually circulates among asymptomatic animals (mammals and ticks) in an enzootic cycle. CCHFV has been detected in many tick species, but *Hyalomma* spp. ticks represent the main viral reservoir, and both transstadial and transovarial transmission occur in this genus^2^. CCHFV causes severe disease in humans, with reported case fatality rates ranging from ~5% to as high as 80% in different countries^1^. To date, there is very limited knowledge available regarding the biology and pathogenesis of CCHFV due to the requirement for the virus to be handled in high-containment laboratories^1^. In recent years, research programs have focused on understanding the virus-mammalian host cell interaction to gain an overview of the molecular pathogenesis of CCHFV^3^. Previously, we demonstrated that there is an interplay between CCHFV and the apoptosis process in mammalian cells^4^. Interestingly, we found that the CCHFV nucleoprotein (N) contains a proteolytic cleavage site, DEVD (a caspase-3 cleavage site), which is conserved in all CCHFV strains^5^. Furthermore, we found that DEVD cleavage inhibits the yield of progeny virus^5^. This finding raised the question of why the DEVD motif has been conserved during evolution of this RNA virus despite substantial genetic diversity among CCHFV strains. This question might be answered by studying the replication of CCHFV in its natural host:ticks. The requirement for the virus to be handled in high-containment laboratories, added to the difficulty in manipulation of infected ticks in a biosafety level (BSL)-4 facility, has made this task challenging^6,7^. To shed light on the role of the DEVD motif in CCHFV replication, we have developed an in vitro tick cell culture model based on a previous observation that tick cell lines can be infected with CCHFV^8^. First, we characterized CCHFV replication in the *Hyalomma anatolicum*-derived cell lines HAE/CTVM8 and HAE/CTVM9^9^ by evaluating viral progeny release, the yield of intracellular viral RNA, and N expression. HAE/CTVM8 and HAE/CTVM9 cells (2 × 10^6^) were seeded in sealed, flat-sided culture tubes (Nunc, Thermo Fisher Scientific) at 32 °C and grown for 48 h and then infected with the CCHFV IbAr10200 strain^10^ at multiplicities of infection (MOI) of 0.1 and 1.0, in 1 mL of culture medium. After 1 h, cells were washed with PBS and cultured in 2.5 mL of complete medium. In studies of the kinetics of viral progeny release, 200 µL of supernatant medium were collected, as indicated in Fig. 1a, for viral titration on Vero cells, as previously described^10^, and an equal volume of fresh medium was replaced in the culture tubes.

**Fig. 1:**
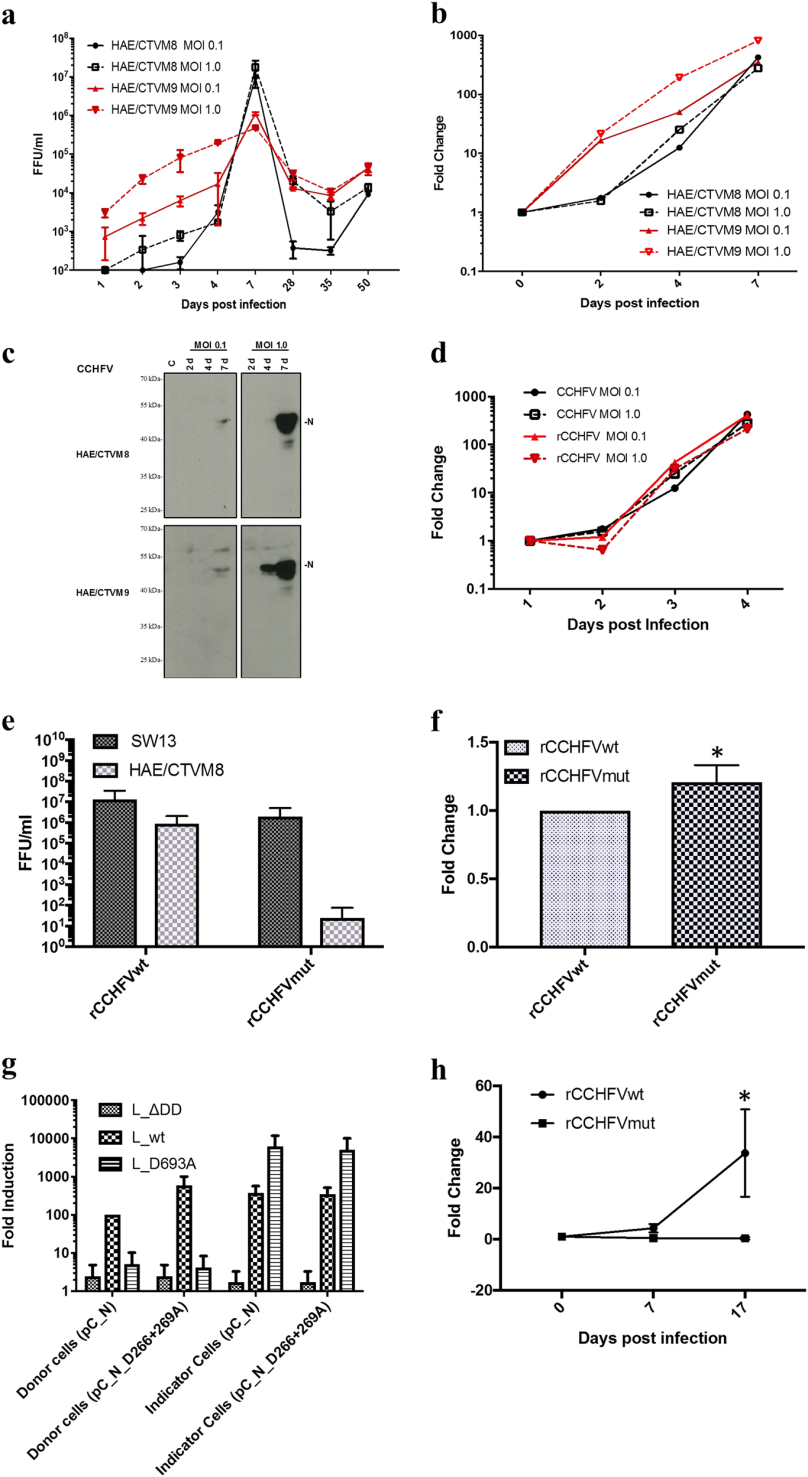
Replication of CCHFV in *Hyalomma*-derived tick cell lines. **a**–**c** Tick cell lines HAE/CTVM8 and HAE/CTVM9 were infected with CCHFV at MOI 0.1 or MOI 1.0. At the indicated time points: **a** Infectious viral particles released were titrated by the focus forming unit (FFU) assay in Vero cells, error bars = S.D.; **b** The relative increase in viral RNA in the infected cells was evaluated by qRT-PCR; **c** expression of the CCHFV-N protein was evaluated by western blot, C = uninfected cells. The experiment was performed three times in duplicate, and sample analyses were performed in duplicate. **d** HAE/CTVM8 cells were infected with the CCHFV IbAr10200 strain or rCCHFVwt at MOI 0.1. Viral replication was compared by measuring the relative amount of viral RNA in the infected cells at the indicated time points. **e**–**f** Human SW13 and tick HAE/CTVM8 cells were infected with rCCHFVwt or rCCHFVmut at MOI 0.1; viral replication was evaluated by: **e** titration of the virus progeny for both cell lines, and **f** measuring the relative amount of viral RNA in the infected SW13 cells. *P < 0.05, unpaired *t*-test. The experiment was performed three times in duplicate, and sample analyses were performed in duplicate. **g** HuH-7 (JCRB0403) donor cells were transfected with the CCHFV tc-VLP system, using either a wild-type (pC_N) or a mutant (pC_N_D266+269A) N, in the context of a wild-type (L_wt) or a transcriptionally incompetent polymerase (L_D693A). An inactive polymerase (L_∆DD) was used as a control. Minigenome luciferase activity was measured 3 days post transfection. The presence of tc-VLPs in donor cell supernatants was detected by transfer of supernatants onto HuH-7 indicator cells expressing L-wt and N and measurement of luciferase activity 24 h p.i. The results were normalized against the control L_wt+N_wt value set to 100%. The experiment was done in triplicate. **h** Replication of rCCHFVwt and rCCHFVmut was also evaluated in HAE/CTVM8 cells over 17 days by measuring the relative amount of intracellular viral RNA. *P < 0.002, ANOVA. The experiment was performed three times in duplicate, and sample analyses were performed in duplicate

Although both *H. anatolicum* cell lines were permissive to CCHFV infection, the kinetics of viral replication differed between them. We found that CCHFV grew faster in HAE/CTVM9 compared to HAE/CTVM8 at the early time points; however, at later times post-infection (p.i.), the yields of progeny virus were comparable between these cells (Fig. 1a). At 7 days p.i., CCHFV-infected tick cells were sub-cultured, and viral infection was monitored by viral titration or RT-PCR for 282 days p.i. Although the sub-culturing of cells complicated the interpretation of viral particle production (Fig. 1a), overall, our data indicated the establishment of a persistent infection (Fig. 1a and Supplementary Table 1).

To evaluate the intracellular viral RNA yield by quantitative real-time Reverse Transcriptase-PCR (qRT-PCR), CCHFV-infected cells were collected, washed with PBS and lysed using the TRIzol LS reagent (Invitrogen). Total RNA was extracted using a Direct-zol™ RNA MiniPrep Kit (Zymo Research) and CCHFV RNA was detected using a RealStar® CCHFV RT-PCR 1.0 kit (Altona Diagnostics) following the manufacturers’ instructions. The amount of viral RNA was normalized to the expression of the putative translation elongation factor EF-1 alpha/Tu (EF1A) gene of *H. anatolicum* tick cells (primers available on request) and expressed as the fold change with respect to the initial virus inoculum (set to 1 at day 0, Fig. 1b) using the ∆∆Ct method for relative quantification of RNA^11^. The results showed that viral RNA increased over time and was more abundant in HAE/CTVM9 cells at the early time points (Fig. 1b). To evaluate viral protein expression, cells were collected and washed in PBS by centrifugation at 335 rcf for 7 min at 4 °C and then processed for western blotting analysis as previously described^4,5^. The level of N protein expression was MOI-dependent and was very high at MOI = 1.0 at 7 days p.i. (Fig. 1c). In HAE/CTVM8 cells, the expression of CCHFV-N was delayed in comparison to that in HAE/CTVM9 cells (Fig. 1c).

Overall, our results showed that CCHFV replicated faster in HAE/CTVM9 cells than in HAE/CTVM8 cells; however, at day 7, the results were comparable between the two cell lines.

These results could be due to the heterogeneity between HAE/CTVM8 and HAE/CTVM9. In fact, all tick cell lines are phenotypically and genotypically heterogeneous, having been derived from the tissues of multiple embryos of individual ticks, as reflected in their light microscopic morphologic appearance^8,9^.

We then used our infection model to investigate the importance of the DEVD motif for CCHFV replication in tick cells. As highlighted above, we previously demonstrated that the N protein can be cleaved in mammalian cells by pro-apoptotic caspase-3 enzymes at the level of a highly conserved DEVD motif, producing two polypeptides of approximately 30 and 26 kDa^5,12^. Using caspase inhibitors, we found that cleavage of CCHFV-N affected the yield of progeny virus and that N protein expression could suppress the induction of apoptosis^4^. Thus, this phenomenon could represent a host cell immune defense mechanism against CCHFV infection^5^. Interestingly, we could not detect such cleavage in tick cells, as western blotting revealed a single ~50-kDa N protein (Fig. 1c). As CCHFV persistently infected these cell lines, and considering the absence of detectable virus-induced cell death, it is most likely that the virus efficiently inhibits apoptosis in tick cells. However, we cannot exclude the possibility that caspase-3 in these cells does not recognize the DEVD site.

To further investigate the function of the DEVD motif in CCHFV replication, we generated recombinant mutant CCHFVs by the previously reported rescue system^13,14^. The wild-type DEVD sequence (rCCHFVwt) was changed to a caspase cleavage-resistant AEVA sequence (rCCHFVmut) by site-directed mutagenesis. After two steps of viral amplification in SW13 cells (ATCC® CCL-105™), the N protein coding sequences of the wild-type and mutated recombinant viruses were verified by nucleotide sequencing (data not shown). To evaluate the ability of rCCHFVwt to replicate in tick cells, we compared by qRT-PCR the kinetics of rCCHFVwt and parent CCHFV IbAr10200 strain replication in HAE/CTVM8 cells, and the observed trends were similar (Fig. 1d). Then human SW13 and tick HAE/CTVM8 cell lines were infected with rCCHFVwt and rCCHFVmut at MOI 0.1. Virus replication was evaluated by qRT-PCR of intracellular viral RNA and virus progeny titration. At 72 h, the titer of the rCCHFVmut in SW13 cells was approximately ten times less than that of rCCHFVwt (Fig. 1e), whereas intracellular rCCHFVmut RNA yield in SW13 cells was 1.3 times greater than that of the wild type (Fig. 1f). To investigate the DEVD motif-sensitive virus replication step, we took advantage of our CCHF transcriptionally competent virus-like particle (tc-VLP) system developed in mammalian cells^15^ that allows discrimination of the transcription and the replication steps. As we already showed using a minireplicon system in BSR-T7/5 cells^12^, we confirmed using the tc-VLP system in HuH-7 cells that mutation of the DEVD site increases transcription. Indeed, in HuH-7 cells producing VLPs (donor cells), transcription of the luciferase minigenome by the viral polymerase L_wt was increased in the presence of the mutant (pC_N_D266+269A) N protein (Fig. 1g). However, no major differences were found in HuH-7 cells infected with VLPs (indicator cells), suggesting the absence of an effect on replication or VLP production. We also used a transcription-incompetent, but replication-competent polymerase (L_D693A)^15^ and similarly did not observe any major effects on replication/VLP production. These data suggested that the DEVD motif may have an as-yet undetermined function, but it is not essential for virus replication in mammalian cells.

Strikingly, we found a strong-negative effect of the AEVA mutation in HAE/CTVM8 cells. Although rCCHFVwt was able to replicate in tick cells, rCCHFVmut showed a strong impairment in RNA replication and only ~100 particles were detectable in just one replicate in one of the experiments for rCCHFVmut (Fig. 1e, h). The inability of rCCHFVmut to produce viral progeny and the dramatic reduction in viral RNA (>99% compared to RNA of the wild-type virus) (Fig. 1h) suggested a significant impairment of replication/transcription of the viral genome that could be due to a malfunction of the N protein in the tick intracellular environment or the inability to interact with one or more key cellular factors required for viral replication. To date, there is a lack of molecular tools for tick cells, such as mini replicon and VLP systems, such that we cannot pinpoint the exact mechanism of function of the DEVD motif. However, our data suggest that the DEVD motif has an essential role in CCHFV replication in tick cells.

In conclusion, our results support the applicability of tick cell lines to studying the biology of CCHFV in vector cells and virus/vector interactions. Processing of the N protein appears to have a moderate effect on viral replication in mammalian cells, but the dramatic inhibition of CCHFV replication after mutation of the DEVD motif in tick cells raises an interesting question about the function of this viral protein in the context of the vector. Targeting the DEVD motif could be a strategy to counteract infection in ticks to reduce viral persistence in the environment. The virus/tick cell culture system reported here provides the basis for further studies to characterize the tick cellular response to CCHFV infections and to determine the mechanism by which tick cells can tolerate persistent viral infections.

## Electronic supplementary material

Table 1
