# Supplementary material for: The DEVD motif of Crimean-Congo hemorrhagic fever virus nucleoprotein is essential for viral replication in tick cells
Source: Emerg Microbes Infect. 2018 Nov 28;7:190. doi: 10.1038/s41426-018-0192-0 (PMC6258742; doi:10.1038/s41426-018-0192-0)
Supplement: Supplementary file 1 — Table 1 [file 41426_2018_192_MOESM1_ESM.docx]

| **Days post infection** | **Detection of CCHFV by RT-PCR** |
| --- | --- |
| 57 | + |
| 73 | + |
| 92 | + |
| 127 | + |
| 212 | + |
| 232 | + |
| 282 | + |

Table 1. Detection of CCHFV in infected tick cells to evaluate the establishment of a persistent infection
